# Agri-silvicultures of Mexican Arid America

**DOI:** 10.1186/s13002-023-00612-5

**Published:** 2023-09-14

**Authors:** Araceli del Carmen Andablo-Reyes, Ana Isabel Moreno-Calles, Beatriz Adriana Cancio-Coyac, Ernesto Gutiérrez-Coatecatl, Alexis Daniela Rivero-Romero, Gerardo Hernández-Cendejas, Alejandro Casas

**Affiliations:** 1 Consejo Nacional de Humanidades Ciencias y Tecnologías (CONAHCYT), Av. Insurgentes Sur 1582, Col. Crédito Constructor, C.P. 03940 Alcaldía Benito Juárez, Ciudad de Mexico Mexico; 2 Escuela Nacional de Estudios Superiores (ENES), Universidad Nacional Autónoma de México (UNAM) Campus Morelia, Antigua Carretera a Pátzcuaro, 8701 Morelia, Michoacán Mexico; 3 Universidad Autónoma de Chapingo. Carretera México-Texcoco, Km 38.5, 56230 Texcoco de Mora, Mexico; 4 Instituto de Investigaciones en Ecosistemas y Sustentabilidad, Universidad Nacional Autónoma de México (UNAM) Campus Morelia, Antigua Carretera a Pátzcuaro 8701, Morelia, Michoacán Mexico

**Keywords:** Traditional agroforestry, Traditional agriculture, Nomadic cultures, Arid and semiarid north of Mexico, Biocultural diversity, Wild and domesticated biodiversity, Geohistory

## Abstract

**Background:**

Agri-silvicultures (ASC) are biocultural practices procuring either the maintenance of wild diversity in predominantly agricultural spaces or introducing agrobiodiversity into forests. In the Mesoamerican region, ASC contribute to food sovereignty and territorial conservation and provide strategies for dealing with global changes. Previous inventories of ASC identified gaps in information about these systems in the Mexican Arid America region. This article raises the general question: How have human interactions between cultural, wild, and domesticated biodiversity in this territory? The particular questions in this paper are: (i) How have historical processes shaped human interactions between wild and domesticated biodiversity in the region? and (ii) What types of agri-silvicultures have emerged in Mexican Arid America since these relationships?

**Methods:**

We trace a methodological border where archaeologists have identified the Mesoamerican region to define our study area as Arid America northern of this line in Mexico. We analyzed agriculturalization processes in Arid America through a historical review. Then, we carry out an inventory of Arid America ASC based on academic papers and other documented experiences. We constructed a spatial database and a typology to understand what kinds of agri-silviculture occur in the region.

**Results:**

We identified several pre-Hispanic agri-silvicultural practices in the region, like hunting, fishing, terraces, gathering, and irrigation systems. The cultivation of native species of maize, beans, and squash even was registered. The Spanish colonization forced the agriculturization in arid northern Mexico, where itinerant hunting-gathering patterns predominated. In the twentieth century, the Green Revolution adopted this area as the principal setting for industrialized agriculture. The industrialized irrigated systems expansion and other political strategies provoked the simplification of productive landscapes. The practices that integrate wild and agricultural diversity systems were marginalized and invisibilized in such a context. Our research group proposes seven types of agri-silvicultural systems (natives agrisilvicultures, the oases agroforestry, Mesquite and Huisache ASC, homegardens and other traditional forms of agroforestry or agri-silvicultures). These agri-silvicultures provide food, medicine, fodder, and other contributions, as income to the families that practice them and protect native and exotic species.

**Discussion and conclusion:**

The agriculturization of the arid environments initiated during Spanish colonization and the subsequent modernizing projects shaped dominant actors and ideologies in the arid north of Mexico. However, aridity has favored ancestral and agroecological relationships between cultures and biodiversity, emerging and subsisting Arid American agri-silvicultures. These agri-silvicultures deserve to be understood, adopted, and adapted to new contexts. They could be essential alternatives in the context of environmental changes.

## Background

Agrisilvicultures (ASC) are the human interactions articulating wild and agricultural biodiversity organized in the context of long-term environmental, social, and cultural complexes and in recent forms arising from continuity and biocultural creativity practiced by dynamic groups of humans in current and changing environmental contexts [1]. In Mexico and other regions of the planet, agri-silvicultures, popular, native, ancestral, traditional agroforestry, ancient agroforestry systems or ethnoagroforestry have been practiced since the origins of agriculture [2–4]. The science of agroforestry has emerged approximately for fifty years, initially named agrisilviculture [5], at first accounting for these forms of human-biodiversity interaction in the world and later taking its cultural position in which it calls itself science-based agroforestry [6]. We propose to use the term agri-silviculture in this work (complexing and culturally and temporarily expanding the term agroforestry) to refer to the cultures of articulating or reconnecting in agricultural environments to forest elements (mainly wild) of plants (perennial, woody, succulent, rosetophilous) but also to other diversities (animals, fungi and microorganisms); and, we are also referring to the integration of cultivated and/or domesticated elements in predominantly wild environments [4, 7–9]. This allows us to integrate the diversity of expressions of these interactions in the Mexican Arid America, including those already recognized by agroforestry science, and expand them from reviewing other fields of knowledge, wisdom, and experiences [1].

In Mesoamerica, systems like pineapple, cocoa, coffee, and vanilla agroforests, base their rationality on cultivated plants that live under the shade of timber, fruit, and ornamental trees, forming agri-silvicultures [8, 10–12]. Milpas include live fences, vegetation islands, isolated trees, and vegetation strips against water and wind erosion. Terraces and semi-terraces agroforestry soften the rugged Mexican landscape, benefiting the systems' capacities to maintain moisture and nutritious soil. Also, hydraulic agroforestry practices beside rivers, ravines, springs, and lakes allow food cultivation in contexts where water excess must be controlled [13]. Family and collective gardens are cultural forms of relationship between wild and cultivated biodiversity, and people are currently practicing these agri-silvicultures in Mesoamerica [1, 8, 12].

Previous studies of agroforestry and agri-silvicultures in arid, semiarid, and subhumid contexts document the contributions of these forms of relationship with diversity and include the generation of economic resources, strengthening food security and sovereignty, satisfying local and global needs, in addition to environmental contributions to mitigate the effects of climatic phenomena, such as frost, drought, rain or atypical wind gusts, in addition to providing shade and protection, forming the habitat of other valuable species, maintaining or increasing soil fertility, favoring its formation and reducing erosion, reducing the effect of non-beneficial insects, increasing the capacity management and control of burning, maintaining hydrological benefits and, in short, constituting important alternatives for mitigation and adaptation to climate change [14–17]. In addition, this relationship is relevant for social learning and the collective creation of knowledge, articulating the worldviews, knowledge, practices, values, and governance of the social units that manage it and the social actors that are interested in them, which include farmers, groups of indigenous peoples and agri-silvicultures, government entities, civil society organizations, and international organizations [4, 18]. However, studies in arid, semiarid, and subhumid lands in Mexico are not prevailing in the literature on agri-silvicultural systems, especially in the portion of Arid America [19].

The delimitation of Arid America and Mesoamerica has been a subject of theoretical-methodological debate among historians, geographers, biologists, ecologists, archaeologists, and anthropologists [20–23]. This work reviews the systems developed in the region called Arid America in the northern portion of Mexico, includes the states of Baja California, Baja California Sur, Sonora, Chihuahua, Coahuila, Nuevo Leon, Durango, Zacatecas, San Luis Potosi, Aguascalientes and the northern part of Tamaulipas, Sinaloa, and Guanajuato [21, 24].

In previous works documenting the diversity of agroforestry systems in Mexico, our research group has identified gaps in information in the country's northern region [1, 4, 8, 12]. In 2013, only two publications were registered for Baja California Sur and Guanajuato. In 2014, with more than 700 publications identified on Traditional Agroforestry Systems in the country, only five referred to the region. In 2016, only two more papers were added to the list, and in 2021, in an effort focused particularly on publications on agri-silviculture in arid and sub-humid zones, the number for the region rose to only 24 publications. Authors explained such scarcity of studies by the greater presence of traditional agricultural systems in central and southern Mexico, while industrialized systems predominate in the north [4]. This general pattern is associated with the original predominance of hunting-gathering patterns over subsistence agriculture in the region; the decrease of native peoples in Arid America due to the particularities of the Spanish conquest and colonization process that involved the submission of these peoples even more brutal than in the south; and to the impulse of the Green Revolution in northern Mexico [4, 12, 25–30]. The Spanish invasion affected the sedentarization and agriculturalization process for the Arid American groups (Chichimecas, Ópatas, Cochimíes, Yaquis, Pápagos, and Series), changing their way of subsistence and their biodiversity relationships. Subsequently, during the twentieth century, the formation of large latifundios, the dispossession of land from indigenous communities, and then industrial agriculture development enhanced in the north deepened the differences between Mesoamerican and Arid American agri-silvicultures [30, 31].

Since 2013, our research group identified specific management systems in the region, Oasis, Tajos, and Mahuechis, that differed from those practiced in Mesoamerica. This study proposes a new approach to understanding the particularities of agri-silvicultural relations in Mexican Arid America, considering that, in addition to the aridity conditions, specific historical processes of the region have led to their decreased presence. This article raises the general question: How have interactions been between cultural, wild, and domesticated biodiversity in this territory? The particular questions in this article are: i) How have historical processes shaped human interactions between wild and agricultural biodiversity in the region? and ii) What types of agri-silvicultures have emerged in Mexican Arid America since these relationships?

## Study area and methods

### Delimitation of Mexican Arid America

Since Paul Kirchhoff proposed an archaeological regionalization to group the pre-Hispanic peoples of the American continent around their cultural coincidences or differences, an intense academic debate has developed on the limits of the region called Mesoamerica and, consequently on the so-called Arid America [32]. Kirchhoff [23] defined Mesoamerica as a cultural area due to its homogeneity and the presence of the "superior" cultivators, who were agricultural and sedentary peoples. He described the Mesoamerican northern border as a diffuse line coinciding with the Sinaloa-Santiago-Lerma and Pánuco rivers. North of that line, the Arid American region grouped the north of Mexico and areas of the southern USA [23].

**Fig. 1:**
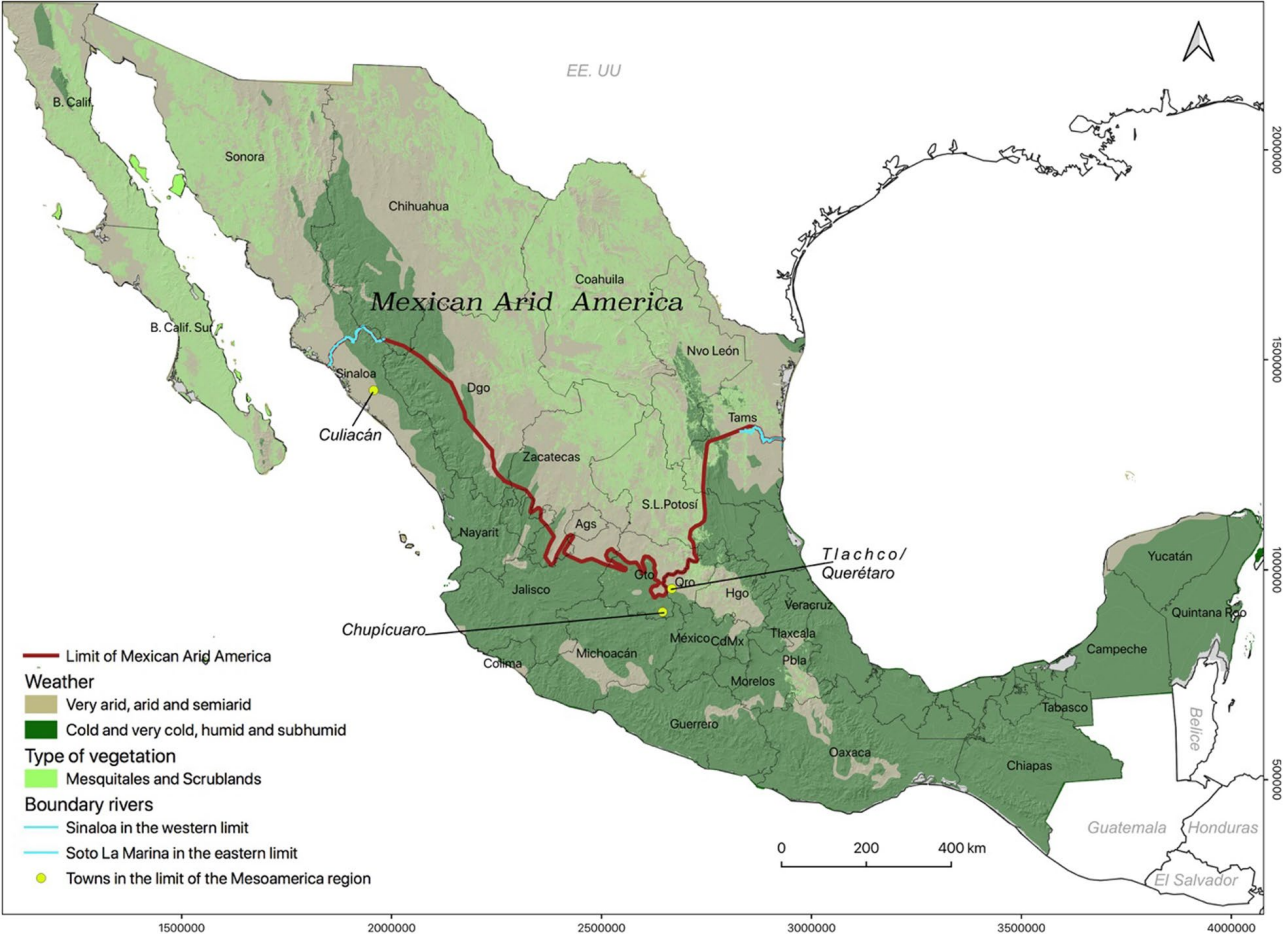
Geohistorical criteria for delimiting Mexican Arid America. Source: Prepared based on [20, 21, 33–41]. Lambert Conformal Conic Projection, Datum WGS 1984

Braniff [20] considered the presence of three elements to identify Mesoamerican cultures: "the evidence of sedentarism (foundations, dumps), agriculture (grains, flat metates) and ceramics…" [20, p. 138]. The northern border of those cultures was dynamic, but our research group considered the limit that was defined at the moment of the Spanish invasion to delimit our study area. Braniff [21, p. 120] mentions that "at the time of the European contacts, Culiacán had reached an urban level" and that Chupícuaro town in the State of Guanajuato was the northern limit of central Mesoamerica. Then, Somohano [33] referred to that in 1536 the area of Tlachco/Querétaro was inhabited by cultivators and was considered the entrance to the Great Chichimeca at the moment of the Spanish Invasion. MacNeish, Piña, and Smith [34–37] found that the Río Soto La Marina in Tamaulipas was the northernmost eastern boundary of the Mesoamerican cultivators. Based on this information [20, 21, 33–37], the maps presented by Braniff and Matos-Moctezuma [21, 24], and following the borderline of semiarid weather [38], we delimited our study area in Arid America in the portion of Mexico comprising the states of Baja California, Baja California Sur, Sonora, Chihuahua, Coahuila, Nuevo Leon, Durango, Zacatecas, San Luis Potosi, Aguascalientes and the northern part of Tamaulipas, Sinaloa, and Guanajuato (Fig. 1).

### Environmental characteristics of Mexican Arid America

This territory has a predominantly arid and semiarid climate on 89.0% of its surface; average rainfall is less than 700 mm per year, and soils are mainly regosols, xerosols, and lithosols. Almost half of the region is classified as mesquital and scrubs, although on the Sierras Madre, there is a significant portion of temperate forests, 13.5%, and tropical dry forests, 3.7% [39]. The altitudinal gradient varies from coastal regions at sea level to altitudes reaching 3200 m above sea level in the Sierras Madre Oriental and Occidental, passing through the depression of the Bolsón de Mapimí in the region of the great Chihuahuan Desert. About these relief conditions, average annual temperatures vary from 17.9 ºC in the warmest areas to 25.3 ºC in the hottest zones, with historical extremes of around 54ºC. These conditions favor the presence of extreme climatic phenomena such as prolonged droughts with annual precipitation averages of only 123 mm, in the case of Baja California, but also floods with historical extremes of precipitation in one day of up to 325 mm. These climatic phenomena also include the occurrence of sandstorms typical of deserts and frosts with historical extremes of up to −15 ºC [42, 43].

The surface area of these entities totals 62.2% of the Mexican territory. In 2020, the region was home to 32.2% of the country's total population, with a density of 33 inhabitants per km^2^. This data is considerably lower than the national average of 64 inhabitants per km^2^ and the Mesoamerican average of 115 inhabitants per km^2^ [41].

In 2020, the Instituto Nacional de Estadística y Geografía (INEGI) registered 70 indigenous languages spoken in the country, 21 of them correspond to native peoples of Arid America: Chichimeco Jonaz, Cora, Cucapá, Guarijío, Huasteco, Huichol, Huichol, Kickapoo, Kiliwa, Kumiai, Mayo, Náhuatl, Otomí, Pa ipai, Pame, Pápago, Pima, Seri, Tarahumara, Northern Tepehuano, Southern Tepehuano, and Yaqui. In the states of Baja California Sur, Nuevo León, Tamaulipas, and Aguascalientes, no native peoples were recorded [41]. Only 9.2% of the population over three years old spoke an indigenous language in Arid America. The data is reduced to 6.5% if we only consider the speakers of the native peoples of the region [41, 44], without including the original people who migrate from the south to the cities and northern agricultural fields.

In 2021, 59.2% of the value of agricultural production and 45.8% of the livestock production at the national level were generated in the 13 states of the Arid American region [45]. Much of this is agroindustrial production, but it is still possible to find traditional agricultural systems.

## Research methods

The general starting point for this paper was to fill the gaps detected in previous research on agri-silvicultures in Mexican Arid America (Table 1) [1, 4, 8, 12]. To understand those gaps, we hypothesize that specific historical processes in the region have led to decreased presence of ASC (Table 1). After this historical analysis, we wonder what types of agri-silvicultures exist in Mexican Arid America, considering that historical processes and aridity have configured particularities. To answer the question, we updated the inventory of publications about ASC in Arid America carried out by our research group [1, 4, 8, 12]. Based on the fieldwork carried out by our team for other research projects in the arid north, we added new keywords in English and Spanish to previous searches to improve the tracing of land-biodiversity practices carried out by northern cultural groups (Table 1). We built a geographic, cultural, ecological, and technical database. Based on the information provided by the documents, we recorded species of flora and fauna and verified the names according to Plants of the World Online (POWO) [46] (Table 2). We registered data on access to government programs and civil society organizations, risks of the systems analyzed, and actions to address climate change and contribute to food sovereignty.

**Table 1:** Synthesis of methods and materials

**Starting point.** Research gaps on ASC in Mexican Arid America **General question:** How have human interactions between cultural, wild, and domesticated biodiversity in this territory?		
Hypothesis	Specific questions	Methods
In Mexican Arid America, the Spanish invasion broke the links between native groups and their biodiversity. Subsequently, the modernization and industrialization processes promoted in the arid north made agri-silvicultural practices invisible	How have historical processes shaped human interactions between wild and domesticated biodiversity in the region?	**Historical analysis** through documentary review
Historical processes and aridity have configured particularities in the agri-silvicultural practices of Mexican Arid America concerning those developed in Mesoamerica	What types of agri-silvicultures have emerged in Mexican Arid America?	**Documentary analysis**: Tracing cases of ASC in academic databases like in Moreno-Calles et al. [1, 8] and other popularization documents like online reports of meetings, congresses, seminars dedicated to rural studies, journalistic notes, and governmental reports in Mexico: Keywords in Moreno-Calles et al. [1, 8] New Keywords: ranches (*ranchos* and *rancherías*), backyard (*traspatio*), rural family (*familia rural*), *ejido*, rural community (*comunidad rural*), land management (*manejo de tierras*), natural resource management (*manejo de recursos naturales*), land use (*uso de suelo*), livestock (*ganado*), and family farming (*agricultura familiar* and *ganadería familiar*)
		**Spatial analysis** Georeferencing and development of a spatial database with ASC cases GIS in QGIS software. Processing of layers of information on physical and social variables of the región. Layers: (1) the Geostatistical Framework and (2) the Main Results by Locality (ITER) from the Population and Housing Census 2020 [43]; (3) Perimeter geographic data of certified agricultural nuclei [47]; (4) Water current [42]; Continuum of Roads [48], information with which a layer of Distance to Superficial Water Currents and another of Distance to Roads was prepared; (5) the Land Use and Vegetation Letter Series 6 [39]; (6) Humidity Ranges [38]; (7) Average Annual Rainfall [49] Development of typology and maps

**Table 2:** Some representative species of each type of Agri-silviculture

Type	Agricultural (species of plants, animals, fungi, and microorganisms, cultivated or domesticated)	Forest/wild (species of plants, animals, fungi, and micro-organisms, wild or under incipient management)
Agri-silvopastoral	Sorghum (*Sorghum bicolor* L. Moench) **, Avena** (*Avena sativa* L.), Maize*(*Zea mays* L.), Bean* (*Phaseolus vulgaris* L.), Squash * (*Cucurbita pepo* L. var. pepo L.H. Bailey), Wheat** (*Triticum aestivum* L.), Coriander** (*Coriandrum sativum* L.), Chili* (*Capsicum annuum* L.), Lettuce* (Lactuca sativa L.), Radish** (Raphanus raphanistrum L.), Tomato* (*Solanum lycopersicum* L.), Caws** (*Bos taurus* L.), Sheep** (*Ovis aries* ssp. aries), Goats** (*Capra hircus* L.), Pigs** (*Sus scrofa* ssp. *domesticus*), Horses** (*Equus caballus* L.), Ducks* (*Anas platyrhynchos domesticus*), Mules** (*Equus asinus* × *caballus*), Donkeys** (*Equus africanus* Heuglin & Fitzinger), Hens** (*Gallus gallus* L.), Turkey* (*Meleagris gallopavo* L.), Swiss chard** (*Beta vulgaris* L), Plum (*Spondias purpurea* L.), Apple (*Malus domestica* (Suckow) Borkh.), Apricot (*Prunus armeniaca* L.)	Mesquite* (*Prosopis laevigata* (Humb. & Bonpl. ex Willd.) M.C. Johnst.), Mesquite* (*Prosopis velutina* Wooton), Mesquite* (*Prosopis glandulosa* Torr.), Huisache/Vinorama * (*Vachellia farnesiana* (L.) Wight & Arn.), Nopal* (*Opuntia sp.*), Guaje* (*Leucocephala glauca* Benth), Maguey bacanora* (*Agave angustifolia* Haw.)
Silvopastoral	Buffel** (*Cenchrus ciliaris* L.), Zacate bermuda** (*Cynodon dactylon* (L.) Pers.), Caws** (*Bos taurus* L.), Horses** (*Equus caballus* L.)	Huisache/Vinorama * (*Vachellia farnesiana* (L.) Wight & Arn.), Mesquite* (*Prosopis laevigata* (Humb. & Bonpl. ex Willd.) M.C. Johnst.), Garambullo* (*Celtis spinosa var. pallida* (Torr.) M.C. Johnston), Guayacán* (*Guaiacum angustifolium* Engelm.), Chapote* (*Dyospyros palmeri* Eastw.), Palo fierro* (*Olneya tesota* A. Gray), Zámota* (*Coursetia glandulosa* A. Gray), Cósahui* (*Krameria erecta* Willd)
Homegardens	Maize* (*Zea mays* L.), Pomegranate** (*Punica granatum* L.), Membrillo** (*Cydonia oblonga* Mill.), Peach** (*Prunus persica* L.), Lemon** (*Citrus aurantifolium* (Christ.) Swingle), Orange ** (*Citrus aurantium* L.), Cilantro** (*Coriandrum sativum* L.), Tomato* (*Solanum lycopersicum* L.), Hens** (*Gallus gallus* L.), Turkey* (*Meleagris gallopavo* L.)	Maguey bacanora* (*Agave angustifolia* Haw.), Maguey* (Agave spp.), Ocotillo* (*Fouquieria splendens* Engelm.), Palm* (*Yucca schidigera* Roezl ex Ortgies), Organ* *(Pachycereus pringlei* (S.Watson) Britton & Rose), Mesquite* (*Prosopis laevigata* (Humb. & Bonpl. ex Willd.) M.C. Johnst.), Pinabetes** (*Casuarina equisetifolia* L.), Mesquite* (*Prosopis laevigata* Humb. et Bonpl. ex Willd)
Milpa	Maize* (*Zea mays* L.), Squash* (*Cucurbita pepo* L. var. pepo L.H. Bailey), Bayo Bean * (*Phaseolus leptostachyus* Benth) Bean* (Phaseolus vulgaris L*.),* Caws** (*Bos taurus* L.), Goats** (*Capra hircus* L.), Donkeys** (*Equus africanus* Heuglin & Fitzinger)	Amaranto* (*Amaranthus hybridus* L.), Amaranto* (*A. palmeri S.* Watson), Amaranto* (A. powellii S. Watson), Nopal* (*Opuntia sp.*), Huisache/Vinorama* (*Acacia farnesiana* (L.) Willd), Mesquite* (*Prosopis laevigata* (Humb. & Bonpl. ex Willd.) M.C. Johnst.), Sage* (*Salvia* L.), Garambullo* (*Myrtillocactus geometrizans* (Mart. ex Pfeiff.)), Pitayo* (*Stenocereus pruinosus* (Otto ex Pfeiff.) Buxb), Ocotillo* (*Fouquieria splendens* Engelm.), Organ* *(Pachycereus pringlei* (S.Watson) Britton & Rose)
Oasis	Missionary olive** (*Olea europea* L.), Missionary grape** (*Vitis vinífera* L.), Quince** (*Cydonia oblonga* Mill.), Higo prieto** (*Ficus carica* L.), Coco** (*Cocos nucifera* L.), Tepari* (*Phaseolus acutifolius* A. Gray), Caws** (*Bos taurus* L.), Date palm (*Phoenix dactylifera* L.), Fig** (*Ficus carica* L.), Lemon** (*Citrus aurantifolium* (Christ.) Swingle), Orange ** (*Citrus aurantium* L.), Peach** (*Prunus persica* L.), Pineapple (*Ananas comosus* (L.) Merr.)	Chiltepín* (*Capsicum annuum* L.), Garambullo* (*Celtis spinosa var. pallida* (Torr.) M.C. Johnston), Mesquite* (*Prosopis velutina* Wooton), Mesquite* (*Prosopis glandulosa* Torr.), Frutilla* (*Lycium exsertum* A. Gray)
Mesquite and Huisache	Caws** (*Bos taurus* L.), Goats** (*Capra hircus* L.), Bees** (*Apis mellifera* L.)	Chiltepín* (*Capsicum annuum* L.), Mesquite* (*Prosopis laevigata* (Humb. & Bonpl. ex Willd.) M.C. Johnst.), Mesquite* (*Prosopis velutina* Wooton), Mesquite* (*Prosopis glandulosa* Torr.), Huisache/Vinorama * (*Vachellia farnesiana* (L.) Wight & Arn.), Chírahui* (*Acacia cochliacantha* Humb. & Bonpl. ex Willd.), Wereke* (*Ibervillea sonorae* (S.Watson) Greene) Sangre de drago* (Jatropha dioica Sessé), Gobernadora* (Larrea tridentata (DC.) Coville)
Native	Maize * (*Zea mays* L.), Squash* (*Cucurbita pepo* L. var. pepo L.H. Bailey), Tépari* (*Phaseolus acutifolius* A. Gray), Algodón* (Gossypium sp.)	Macuchi* (*Nicotiana rustica* L.), Buli* (*Lagenaria siceraria* (Molina) Standley), Tomato* (*Lycopersicon esculentum* P. Mill.), Chicura* (*Franseria ambrosioides* Cav.), Igualama* (*Vitex mollis* HBK), Pitahaya* (*Stenocereus thurberi* (Engelm.) Buxbaum), Mushroom* ^as^(Okowí Chupíkare, Raramuri name), White water mushroom* (Okowí Ripomi, Raramuri name), Yellow mushroom* (Okowí Sawaroáme, Raramuri name), Water mushroom* (Okowí Sitakame, Raramuri name), Cholugo* (Nasua narica), Venado* (*Odocoileus virginianus*), Octopi steak* (*Hapaj cosni*, Seri name (*Octopus hubbsorum* Berry), Rattlesnake* (*Crotalus* spp.), Suction cup* (Cotopis, Seri name (*Turbo fluctuosus* W. Wood), Large caasol* (Caasol cacat, Seri name (*Ambrosia salsola* (Torr. & A. Gray) Strother & B.G. Baldwin)), Torote* (Xoop, Seri name (*Bursera microphylla* A. Gray), Haaxat Seri Name* (*Larrea divaricata* subsp. *tridentata* (Sessé & Moc. ex DC.) Felger & Lowe)

*Native **Introduced

*Source*: Information obtained from the inventory of items with species listings [16, 84–86, 89–94, 97–99, 101–107, 109]

^a^The authors do not report the fungal species [103]

A spatial database was unified by adding the documented studies up to 2020 in Moreno Calles et al. [1] to the inventoried cases for Mexican Arid America until January 2022. Our research group built a GIS, and we integrated the layer generated by the region cases with other information layers on physical characteristics, land tenure, and the original language-speaking population, among others (Table 1). The layers and maps in the QGIS software in Lambert Conformal Conic projection, Datum WGS 1984, were processed by our study group.

Finally, we developed a typology of the ASC inventoried based on three criteria: the interactions reported between cultural groups and wild and agricultural biodiversity; the particularities resulting from historical processes, represented by Modernization and Green Revolution elements in the agri-silviculture relations; and the practices developed to face or take advantage of the arid lands.

## Results

### The historical process of interactions between human beings and biodiversity in Mexican Arid America

#### Spaniards' invasion

The Spaniards advanced toward Arid America in 1546 [50]. There was a great diversity of ethnic groups in that area as documented by anthropological and archaeological studies [51, 52]. There is no indigenous versions of the history of those people. Eurocentric perspectives misunderstood those cultures as "warrior and primitive tribes" [53] and "barbarians" [52]. European narratives come from the vision of the military and religious who tried to integrate these groups into civilized life.^1^ Some stories of Jesuits and Franciscans graphically describe the "barbarity" of these northern Indians: cruel, dirty, uncultured, cannibalistic, and violent, among many other adjectives accompanied by illustrations about their "diabolical customs"^2^ [55, 56]. The close relationship of the Arid-Americans with the wild environment through hunting and gathering as ways of life contrasted with the Eurocentric vision of civilized humans disconnected from nature [57].

Spaniards directly or indirectly exterminated most of these Arid American tribes [25–27, 58]. Historians documented this process as the "Chichimeca War"; according to Tomé [22], this war lasted a century [59]. In the north, the Spaniards did not find civilizations like the Mexica, Maya, or Purépecha, with complex social organizations, defined settlements and territories, and diversified agriculture [20]. The tribes of the north interacted differently with their lands because the predominant aridity laid restrictions for establishing permanent settlements, and cultivating was not possible as in Mesoamerica [21]. Peoples of the region practiced hunting, fishing, and gathering as the principal mode of subsistence [51, 52, 60].

 The nomadic tribes used many plants for food and medicine [27, 60]. Valdés [52, p. 68]  described the close relationship of the desert nomads with the mesquite: "this plant provided sugars, proteins, minerals, firewood, bread, liquor, gum, and paint." The nomads of the Altiplano of Guanajuato established a similar relationship with the *nopaleras* of the San Luis Potosí and Zacatecas region. Mellink et al. [61] explored the hypothesis that the Guachichiles, one of the Chichimec groups that inhabited the *Tunales* in San Luis Potosí, could have practiced sedentism thanks to the abundance of food provided by the *nopales* and mesquites in their lands [61].

However, other Arid American cultures built complex irrigation systems to manage seasonal or permanent bodies of water. The priest Father Kino wrote in 1699 [62] about the tribes Pimas, Yuma, Opas, and Cocomaricopas inhabitants in the Río Colorado, actually the border between Sonora and Baja California, where the people fed on abundant fishes, maize, beans, and squash. About 150 km to the northeast, archaeologists identified the remains of the Hohokam culture that settled on the Sonoran Desert two centuries before the arrival of the missionaries and whose vestiges left evidence of an advanced irrigation system developed to take advantage of the currents of the Gila River [63]. In Durango and Zacatecas, the Chalchihuite's culture was contemporary to the Hohokam. In that sites have been discovered large settlements whit hillside terraces, pottery, and stone artifacts related to maize agriculture [64].

Álvarez [65] described the case of the Machomoncobe, a culture that inhabited the coastal plain of the Río Mayo in Huatabampo, Sonora, from 177 BC until after 1018 AD. The site records contain evidence of the diversity of annual subsistence practices of its inhabitants. The cultivation of maize (*Zea mays* L.), common bean (*Phaseolus vulgaris* L.), and native *Tépari* bean (*Phaseolus acutifolius* A.Gray) complemented the activities of hunting, fishing, and gathering of vegetables and mollusks in the diverse ecological systems to which this group had access. Álvarez [65] concludes that in the last phases, the settlement achieved more permanence but with short movements of its inhabitants.

**Fig. 2:**
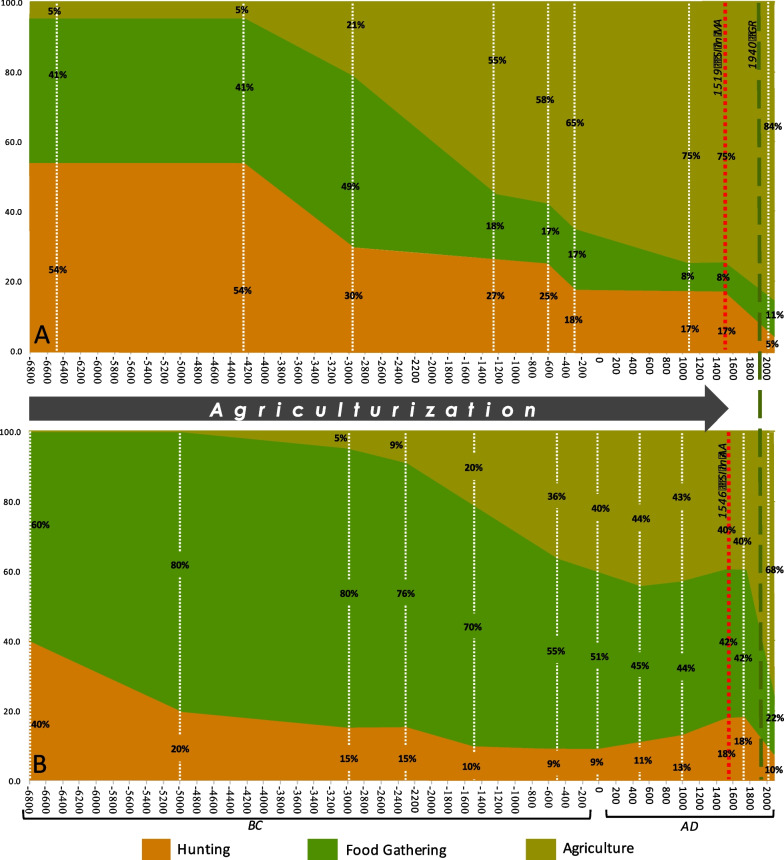
Changes in the composition of the human diet in Tehuacán, Puebla and on the southeastern border of Mexican Arid America (Tamaulipas). SI: Spanish Invasion; MA: Mesoamerica; AA: Arid America. Source: Part** A**: based on MacNeish [35] up to 1500 AD; and for the last date in 2022, based on data from Casas et al. [70]. Part** B**: based on the estimates of the composition of the diet made by MacNeish [34] in the sierra of Tamaulipas in the southeast of the state, up to 1758; and as a reference to the current composition of the diet of the native population in Mexican Arid America, the last date for 2022 correspond to the Raramuri cultural group, a native people of the Sierra Madre of Chihuahua [70]

Based on MacNeish's research [34, 35], we compared the changes of human diet between Mesoamerican and Arid American groups. Part A of Fig. 2 shows the composition of the human diet in the Tehuacan Valley in pre-Hispanic times, between 6550 BC and 1070 AD. MacNeish [35] estimated for that region of Mesoamerica, at least 75% of their people's diet depended on cultivation at the time of the Spanish invasion. For Arid America, there are no such detailed studies on inhabitants' diets. However, MacNeish [34] carried out archaeological research in the southeast of Tamaulipas, near the Soto La Marina River, whose results could be representative of the Mexican Arid America groups. MacNeish [34] estimated that agriculture in the mid-sixteenth century contributed 40% of the diet of the ancient towns of Tamaulipas. People in the region practiced agriculture from approximately 3000 BC. In Chihuahua and Sonora, archeologists identify the first farmers between 2500 and 1500 BC [66, 67]. Part B of Fig. 2 highlights the importance of agriculture/horticulture, gathering, and hunting for the subsistence of the original populations before the Spanish invasion. In Mesoamerica, agriculture had been developed for more than 8000 years [35, 68], while in Mexican Arid America, only 4000 years and limited to more restricted sites [34].

The Mexican Arid American groups' extinction or "reduction" probably limited the agricultural knowledge in arid regions [69]. The advance of the Spanish invasion into northern Mexico was a turning point for those groups in terms of their relationship with biodiversity, but also for those who later inhabited Mexican Arid America. Spaniards, Creoles, mestizo groups, and even the Mesoamerican ethnic groups, allied with the Spaniards in the conquest of Arid America, had to agriculturalize the desert and impose a new way of inhabiting it.

The red line (Fig. 2) highlights the links preserved or built after the Spanish Invasion between Arid American people with the wild diversity, either as a cultural heritage of the original ethnic groups or co-produced by the new cultural groups that inhabit the region. In 1767, the expulsion of the Jesuits closed the first chapter of colonization in northern Mexico. The next episode came with the Bourbon Reforms at the end of the eighteenth century. Although the effect of the reforms was brief, they laid the foundations for land redistribution in the region during the following phases of colonization. Evangelization, war, epidemics, and miscegenation undermined the resistance of most of the native ethnic groups; only a few ethnic groups in the northwest continued in latent rebellion: Yaquis, Mayos, Opatas, Pimas, Seris, Comanches, and Apaches [25, 26, 54]. This resistance was a response to the privatization policies and the breakdown of communal organization and production practices that had been somewhat tolerated and promoted by the Jesuits.

#### Mexican independence and revolution

During the first half of the nineteenth century, the main concern in the northwest, more than the independence revolution, was the guerrilla warfare led by the Indian rebels [25, 26, 71]. The northern intendancies participated tangentially in the conflict. Most of their governors ultimately signed the *Plan de Iguala* in 1821 and joined independent Mexico [72]. For the northern military in power, this revolution represented an opportunity to consolidate their large estates and privileges.

After the triumph of the liberals in 1867, the next wave of colonization took shape through the Reform Laws, with the disentailment of church's properties and lands considered uncultivated. For this purpose, the government creates *Compañías deslindadoras,* boundary-marking companies, reconfiguring land tenure in northern Mexico, mainly in Sonora and Chihuahua [73]. This process reduced the indigenous territories preserved after the secularization of the Jesuit missions and intensified the struggle of the northern ethnic groups for their lands^3^ [25].

Boundary-marking companies' creation was part of a political and economic project. That plan aimed to link some regions of Mexico to emerging international markets, which in turn implied creating vast extensions of irrigated agricultural areas, as was the case in the Comarca Lagunera, the regions of the Yaqui River, the Mayo River, and a good part of northern Sinaloa [31, 73]. In the Yaqui region was a very violent process of dispossession of land and water by the government and the Mexican army. It was an episode that could be considered a second policy of extermination and genocide of the Yaqui people. It ended in 1908 with the banishment of thousands of Yaquis to be sold and enslaved in the henequen fields in Yucatán [26].

In northern Mexico, a model of land ownership was consolidated characterized mainly by the formation of large *latifundios*^4^ in San Luis Potosí, Coahuila, Zacatecas, and Chihuahua and linked to the foreign market driven by the policy of promoting international trade that was part of the modernizing project of the *Porfiriato* [74].

Large landowners, the Sonoran military, the Northern Division, and the Yaqui and Mayo ethnic groups carried out the Mexican Revolution in the north [26]. In 1934, Lázaro Cárdenas won the presidency of Mexico. He promoted the reconfiguration of land tenure through land distribution. The dismantling of the *latifundio*s and the creation new *ejidos* were essential government policies. In five years (1935–1940), the number of agrarian nuclei doubled and allowed the formation of new actors for the following project of the industrialization of the Mexican countryside [75].

### Green revolution

In 1940, the Rockefeller Foundation focused its interest on Mexico. That company and the Mexican Government began a large experimentation project in Sonora with maize (*Zea mays* L*.*) and wheat (*Triticum aestivum* L*.*) to increase their productivity and resistance to pests. This plan started what has been called the Green Revolution [29]. Rockefeller Foundation developed this project mainly in the Yaqui Valley and extended to northern Mexico. The modernization of Mexican agriculture from 1940 onward transformed the aridity through hydraulic works that opened large land extensions to cultivation, whose productivity was detonated based on the principles of the Green Revolution: improved seeds, fertilizers, and pesticides [29]. Sixty percent of the investments in irrigation works made between 1941 and 1970 were concentrated in Arid America [30, p. 101].

This foundation also promoted a livestock modernization project. Two elements constituted the essence of this project. The first was the *Programa Nacional de Desmontes* (PRONADE) promoted by president Luis Echeverría in 1972, which in its first stage alone, planned "the clearing of 320,325 ha of new lands, 222,000 for livestock activities"^5^ [76, p. 123], among these lands were at least 70,000 ha in the northern states. The second was the introduction of buffel grass (*Cenchrus ciliaris* L.) in the 1940s as a panacea to increase the productivity of pastures in scrub and mesquite areas [77, 78]. In Sonora, the cradle of the G.R., the size of cultivated and induced pasture in Sonora has multiplied more than four times from 1985 to 2014 [39].

The results of the modernization project simplified the productive landscapes, and the ancestral knowledge was blurred. Arid American lands took a new way of relating to biodiversity. Nowadays, northern Mexico is an example of dominion and control over nature. It has seen the birth of the Desert Agro-titans, corporate agriculture, and livestock production [79].

The historical review highlights why agri-silvicultures development in the northern cultures has been different concerning Mesoamerica. From the climatic challenge itself to the historical events that restricted the domestication of native species and have hindered the conservation of the relationship with wild species. However, agri-silvicultures are persistent. People find a way to relate to their spaces, to inhabit them from their cultures, and to flow with the possibilities they offer them. In Mexican Arid America, its inhabitants face drought, heat, extreme rains, frosts, and sandstorms, which often destroy their spaces, but tolerate and continue; seeking strategies, diversifying, and taking advantage of the arid elements [42, 43, 80, 81], even importing components from the agro-industrial systems[94, 95].

## Agri-silvicultures in Mexican Arid America

**Fig. 3:**
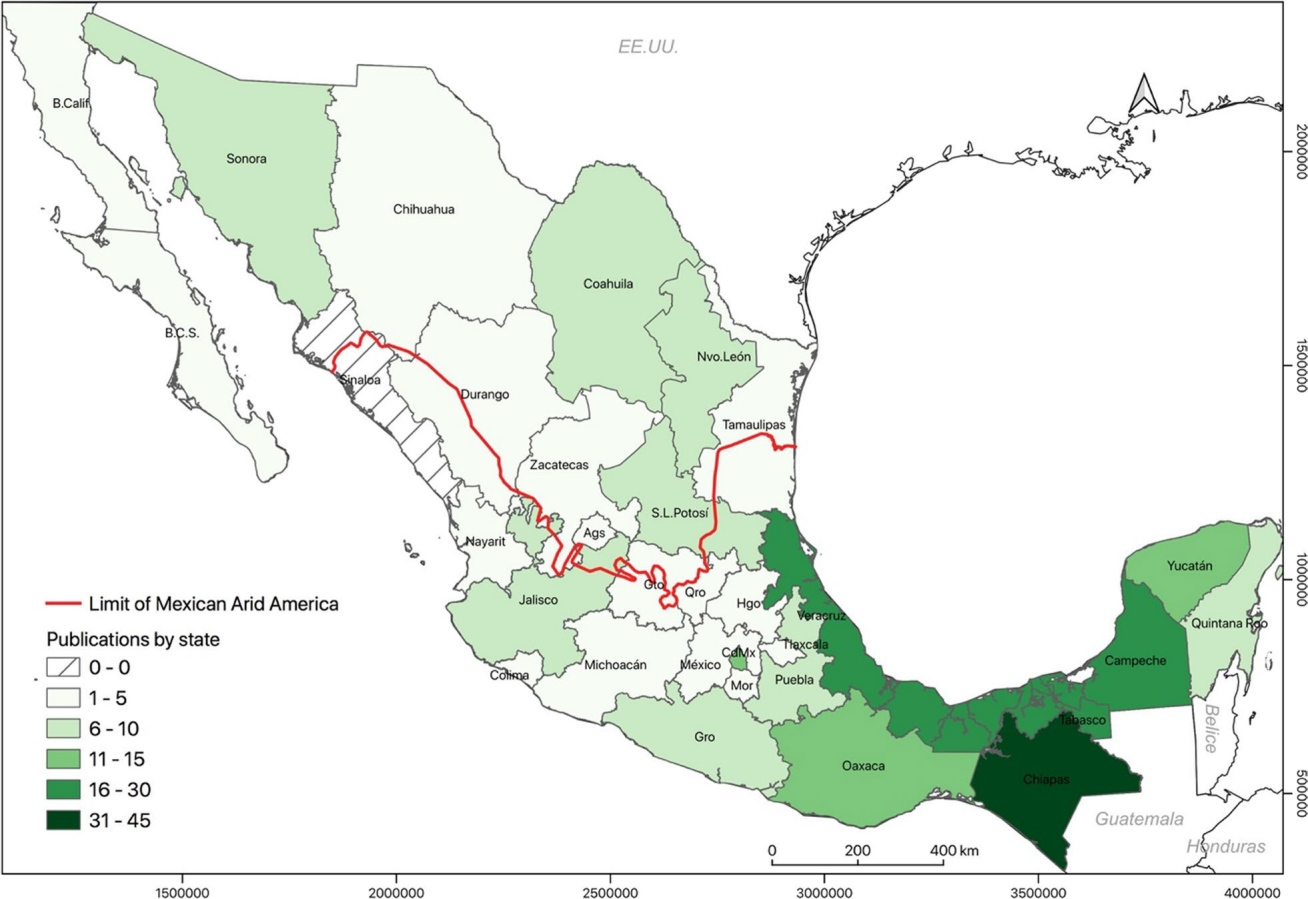
Geographical distribution of publications about agri-silvicultures in Mexico, updated to 2022. Source: Based on the inventory of agri-silvicultures for Mexico. Lambert Conformal Conic Projection, Datum WGS 1984

Our group prepared the database on agroforestry studies for Mexico in 2022, included 730 studies. Only fifty were carried out in Mexican Arid America (6.8%). The spatial distribution of the inventoried publications is in Fig. 3. The map shows the concentration of studies in southeastern Mexico.

**Fig. 4:**
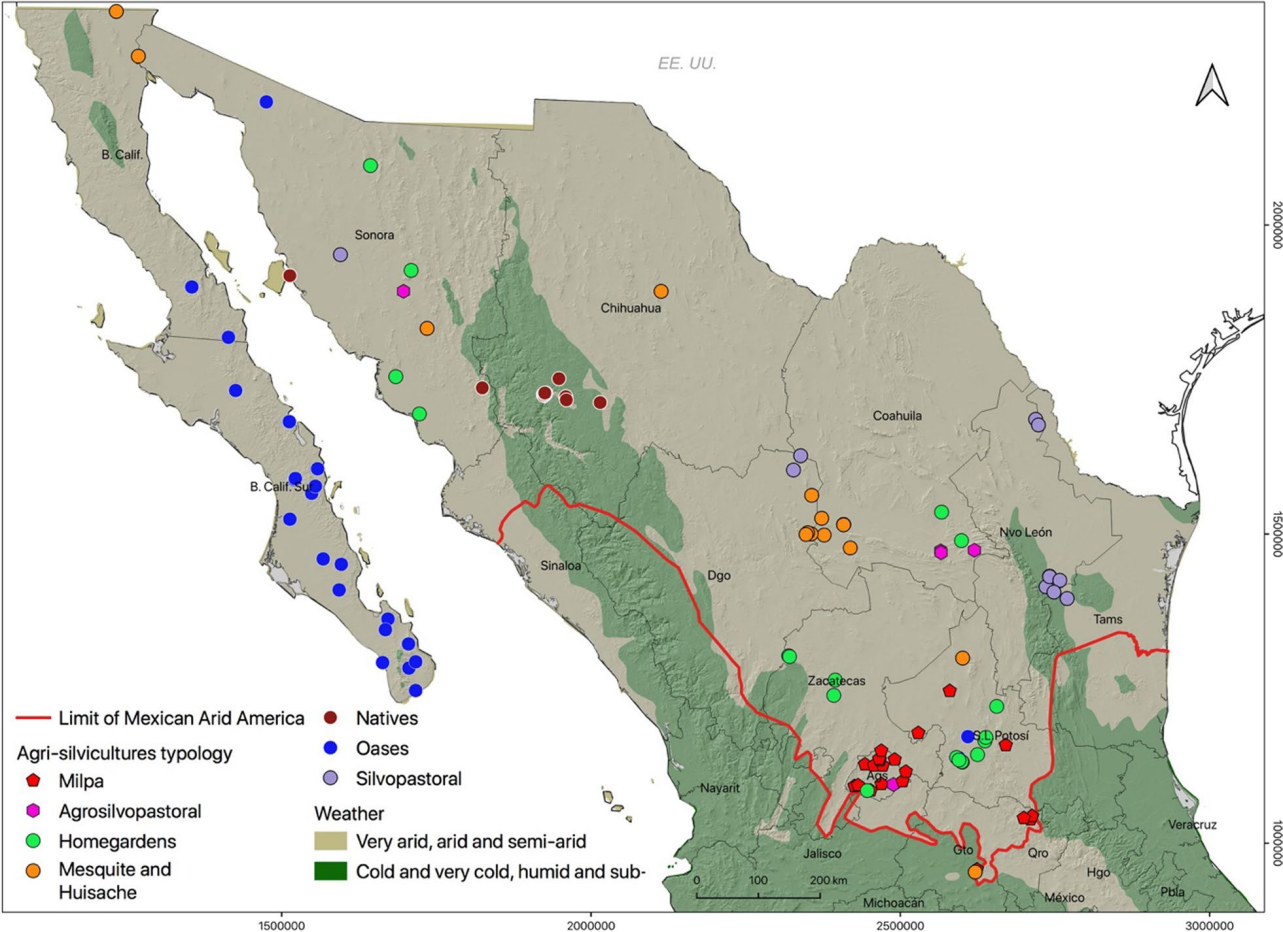
Typology of Agri-silvicultures in Mexican Arid America. Source: Based on the inventory of Agri-silviculture for Mexican Arid America. Lambert Conformal Conic Projection, Datum WGS 1984

The database on ASC studies included articles, book chapters, a report, and a journalistic note. These documents describe 118 places where agri-silvicultures in Mexican Arid America exist. The number of cases in Baja California Sur, Chihuahua, Aguascalientes, Coahuila, and San Luis Potosí stands out. More publications are for Coahuila, Sonora, and Nuevo León states (Fig. 4).

### Typology of agri-silvicultures in Mexican Arid America

The analysis of the history of agri-silvicultures in Mexican Arid America and their interactions with aridity allowed us to propose a more detailed typology: Agrosilvopastoral, Silvopastoral, Homegardens, Milpa, Oasis, Mesquite and Huisache, and Natives. The first four categories correspond to traditional forms of agroforestry systems [3, 82] but with particularities that we highlight in every case. The following three (Oasis, Mesquite and Huisache, and Natives) describe relationships identified as characteristic of Mexican Arid America due to their specific interactions with situated biocultural diversity (Fig. 4).

**Fig. 5:**
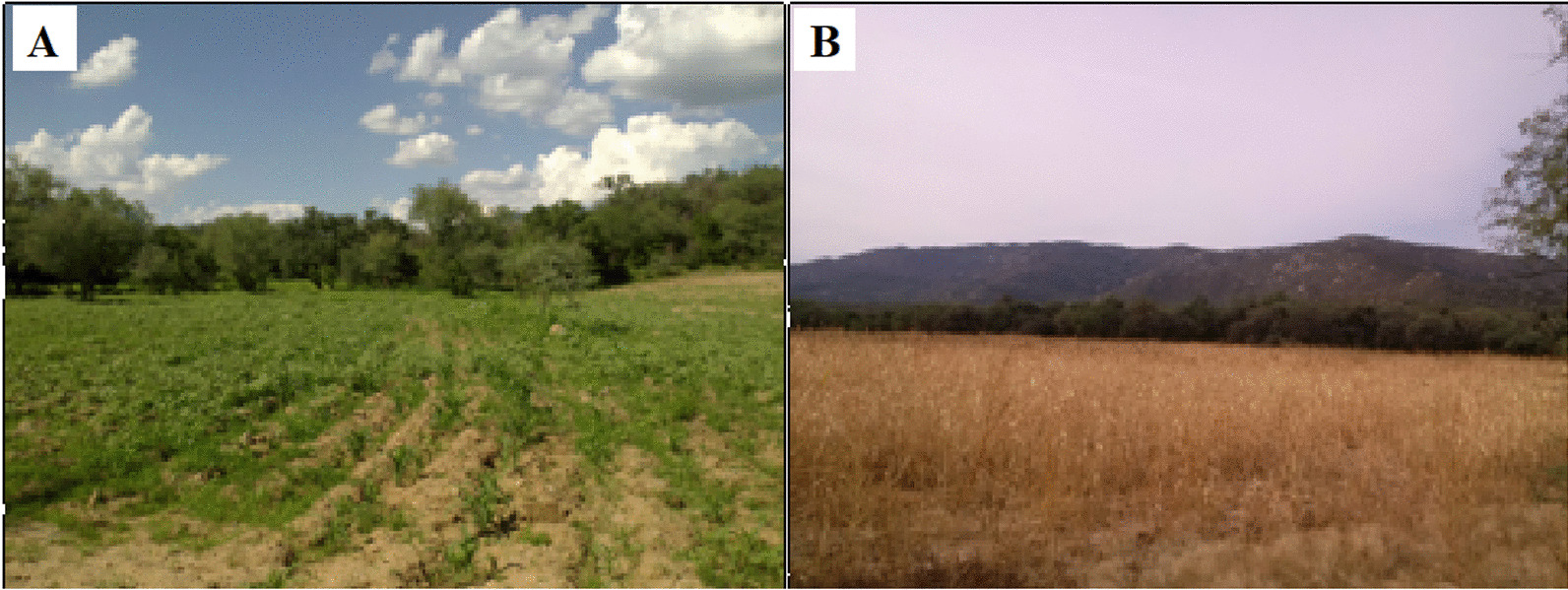
Agri-silvopastoral. **A.** Rainfed maize, in the background mesquite and native vegetation, for dual-purpose cattle feed and fresh cheese production in Cobachi, Sonora, Mexico, August 2013. **B** Rainfed forage sorghum, in the background mesquite, for dual-purpose cattle feeding and fresh cheese production in Pueblo de Álamos, Sonora, Mexico, November 2014. Photographs by Araceli Andablo

We defined agri-silvopastoral types as the relationship promoted by cultural groups between wild and cultivated diversity, and livestock (Fig. 5). This system takes advantage of different spaces, the plots and backyards, to grow fruit trees, vegetables, fodder and maizefield crops, poultry, and other domestic animals, and the pastures to take advantage of, protect, and promote wild or introduced fodder, to raise livestock. In these areas, food produced for humans competes with food intended to feed livestock. The case of maize is illustrative; it has gone from human food to fodder. Families decide about the vocation of their spaces by the need to integrate into the agroindustrial system through some intensive practices such as cattle stabling to speed up fattening and obtain good-weight calves for sale [83]. Figure 5B shows in Sonora state a combination of *mesquites*, *vinoramas*, *chírahuis*, and other native trees that provide forage, with cultivated sorghum in a plot that years ago was used to grow maize and squash (see Table 2 for scientific names).

In Coahuila, agriculture is seasonal, and the main food crops are maize and beans. Families introduced forage such as sorghum, oats, and wheat to make silos for the dry months; in the backyard, they grow squash, coriander, chili, lettuce, radish, *nopales*, tomato, and chard; and also have fruit trees such as plum, peach, pomegranate, apple, apricot, and lemon (Table 2). The domestic animals that they manage are cattle, sheep, goats, pigs, horses, mules, donkeys, and poultry (Table 2). The principal livestock product is milk, but they also obtain meat and eggs [84].

Coahuila's agri-silvicultures are for self-consumption, and people also sell the surplus. Salaried work in industries close to the urban area complements the income obtained from agriculture. Families estimate that, on average, a quarter of the production goes to the market. One of the principal vulnerabilities that families identify is the loss of agricultural vocation due to migration to cities. In addition, the constant and prolonged droughts typical of the region and pests make production difficult [85].

In Aguascalientes, an experimental study reported the rotation of sorghum and beans with live barriers of nopal and *guaje*. The Instituto Nacional de Investigaciones Forestales*,* Agrícolas y Pecuarias (INIFAP) tested agroforestry strategies for controlling soil erosion in arid conditions with high-intensity and short-duration rainfall [86].

**Fig. 6:**
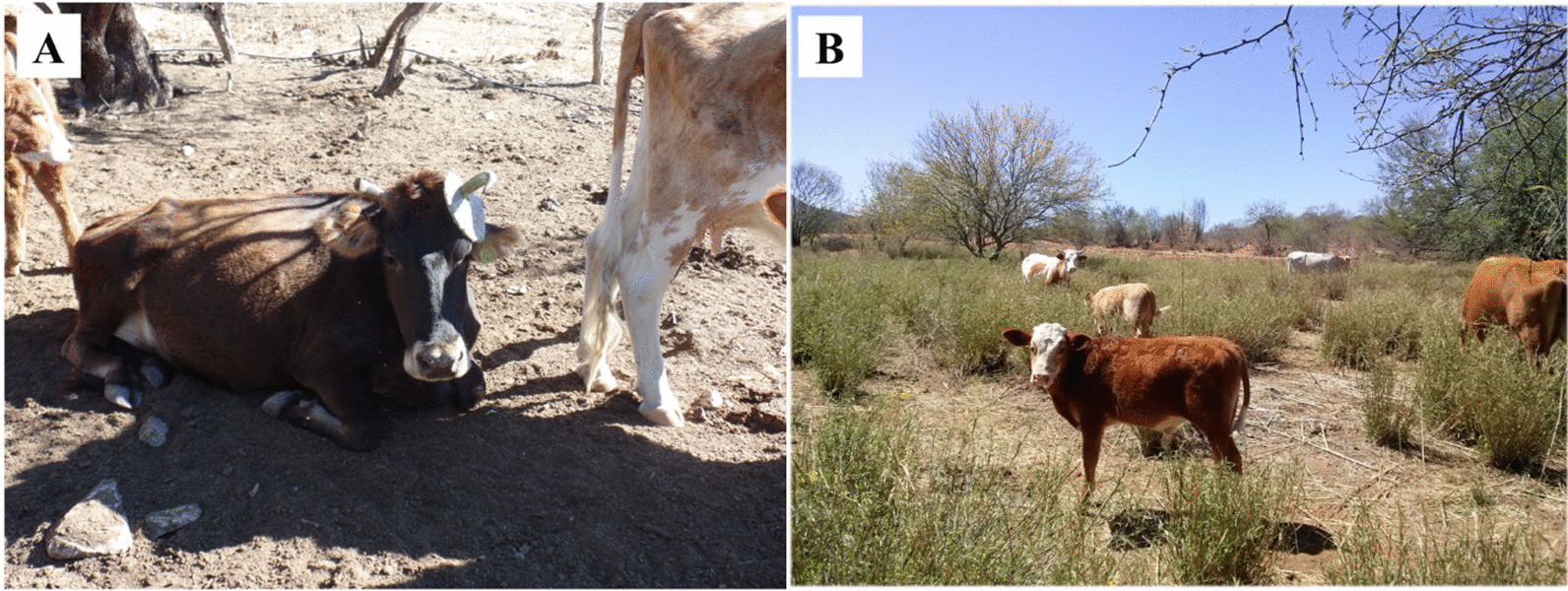
Silvopastoral**. A** Cows feed on nopales in the Sierra Huérfana, Pueblo de Álamos, Sonora, Mexico. May 2016. **B** Meadow with buffel in Tecoripa, Sonora, Mexico. March 2014. Photographs by Araceli Andablo

*Silvopastoral* practices refer to the relationship promoted by cultural groups between wild forage, introduced grasses, and livestock (Fig. 6). The link with local biodiversity is woven through cattle grazing. Spaniards introduced cattle to the Americas, but recently, the modernization process promoted genetic improvement by importing European breeds specialized in meat production. The ASC introduced these breeds into its herds but has adapted them to the arid and semiarid conditions and diversified their production [87]. To take advantage of these environments, they conserve nomadic management practices inherited from colonial times [88]. The pastures introduced by the Green Revolution are part of the strategies in these systems, with undesirable environmental consequences.

We recorded between 13 and 42 species in the grazing sites, with a dominance of the *Fabaceae* family, which contributes to soil regeneration by providing nitrogen. The main species reported are mesquite and huisache [89] (Table 2). In the sites studied, native grasses are essential. Buffel grass exists in the region, an invasive species that reduces native species diversity.

In this group, it is vital to highlight local ancestral management of the "feral or *mesteño*" cattle. Spaniards developed this management in the Bolsón de Mapimí region in the sixteenth century. This area is at the confluence of the States of  Durango, Coahuila, and Chihuahua. Due to the arid conditions that characterize it, 300 mm average annual precipitation, and vegetation microphyllous desert scrub, cattle ranching could not develop as in other places with better possibilities for cultivated or native forage wealth. In the Bolsón de Mapimí, the cattle became feral for two reasons: first, they could not be kept close to the haciendas due to the low pasture (*agostadero*) coefficient. The second argument is the constant irruptions of the native ethnic groups in the area did not allow the ranchers to guard their cattle. In this way, cattle grew freely in the area until it was peaceful at the end of the nineteenth century [88].

In these cattle regions, scholars identified recurrent droughts and the alteration of biodiversity caused by grazing. Thinning of the native vegetation to introduce buffel weakens the regenerative capacity of the diversity due to the low availability of nitrogen in the soil [89]. This introduced species has a great invasive potential to replace native biodiversity, affecting native grasslands and adjacent areas [77, 78]. A positive result reiterated in the case studies is the regenerative capacity of the *Fabaceae* family to face deforestation. Another possible alternative in sites with extreme aridity could be the feral management of livestock to avoid overexploitation of the rangeland.

**Fig. 7:**
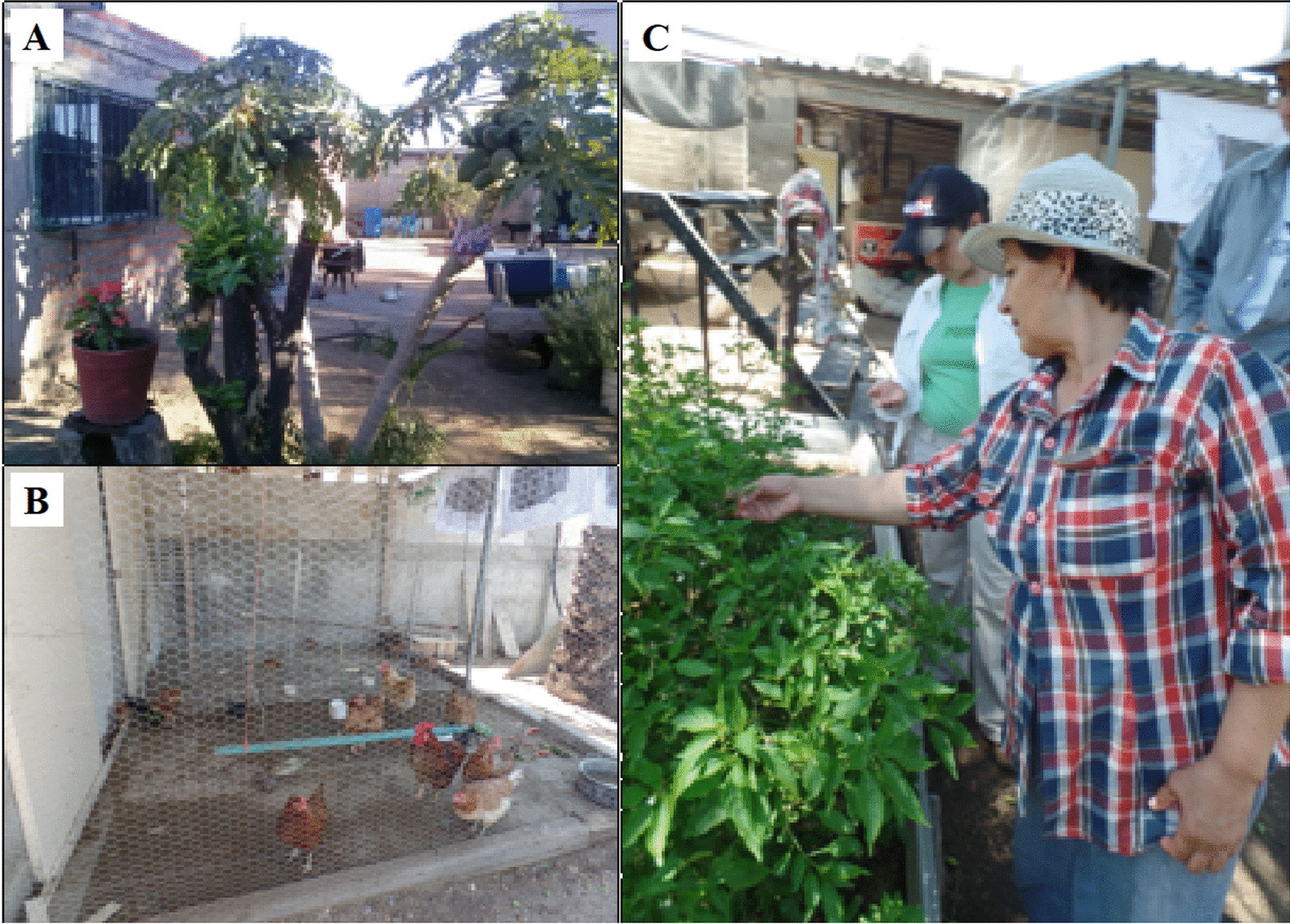
Homegardens. **A–C** From Hilda's Homegarden in Pueblo de Álamos, Sonora, Mexico. July 2018. Photograph by Araceli Andablo

*Homegardens* are identified mainly by their proximity to the family home, where cultivated and wild elements are maintained (Fig. 7). Families manage four to 46 species of fruit trees, vegetables, legumes, agaves, nopales, and other wild species (Table 2). The care given to native species such as *maguey*, mesquites, and cacti promotes their conservation. For example, in Sonora, *maguey* is germinated and grown in the homegardens until it can be transplanted to the plots [90]. Families, too, have domestic animals, cows, sheep, goats, turkeys, donkeys, horses, and hens, to obtain milk, cheese, meat, eggs, and transportation and cargo services [91].

The objective of the homegarden is self-consumption and the occasional sale of surpluses. The families studied reported having income from other activities: employment as day laborers in agricultural fields, in tourism companies, other salaried jobs, sale of cheese, calves, *bacanora*,^6^ migration, and, in the case of two Yaqui communities in Sonora, from renting their land [92, 93].

In arid urban sites, the presence of homegardens contributes to temperature regulation due to the concentration of humidity and shade [94]. Some families have developed strategies to solve problems related to climate and water, such as the reuse of gray water [95]. However, these practices face threats, such as land rental for the establishment of monocultures for export, which causes the loss of diversity and local knowledge about traditional crops; sanitary measures that restrict the presence of domestic animals in the family garden [93]; migration; droughts and frosts; pests and untimely flowering due to climate change; lack of generational replacement [95]. It was also identified that collective or community garden initiatives do not prosper as in the south of the country [96].

**Fig. 8:**
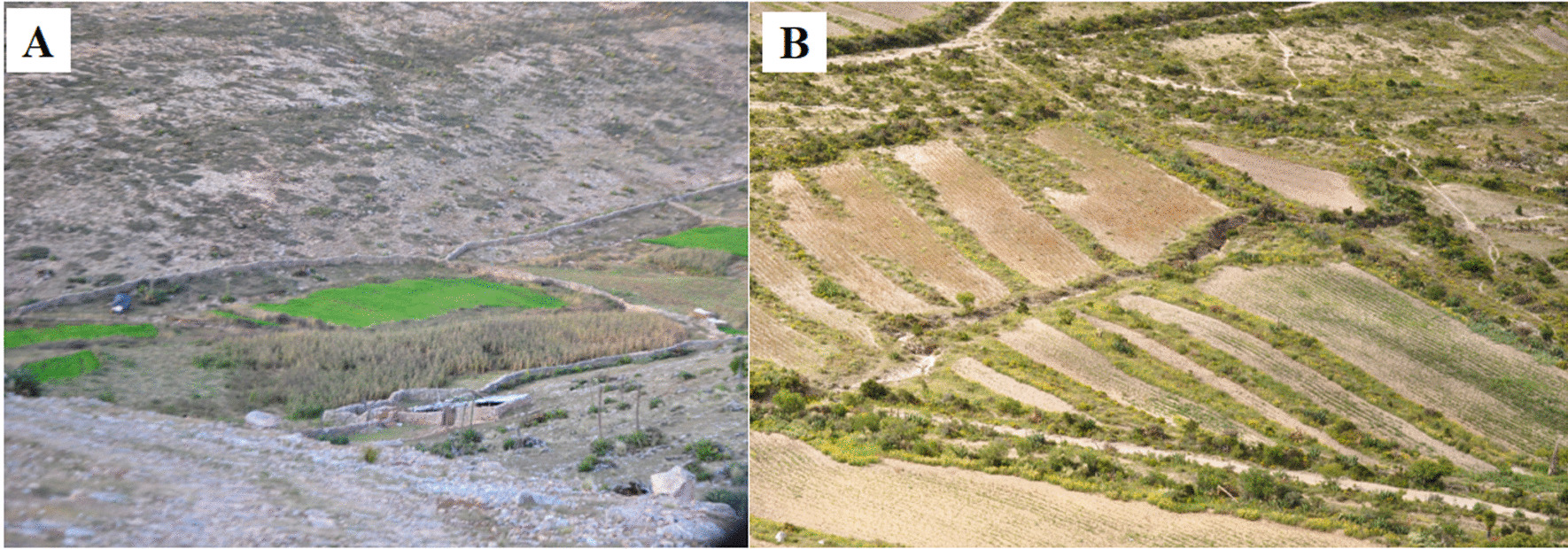
Milpa. **A**, **B** Agricultural plots near Real de Catorce, San Luis Potosí, Mexico, December 2017. Photographs by Gerardo Hernández

*Milpas* are systems where annual crops interact with forestry elements and wild ruderal species (Fig. 8). Families establish a relationship with biodiversity through their plots. These ASC develop soil conservation and recovery strategies in sites prone to erosion and use native species with thorns to build living fences in cultivation areas such as: *ocotillo*, palm, organ, mesquite, and other introduced species such as *pinabete* [97] (Table 2). The predominance of agroindustrial monocultures and politic that support productive specialization threaten the diversity of traditional milpa crops and promote the replacement of food crops with fodder [87].

In addition to cultivating domesticated species, they conserve other arboreal and ruderal species used as food, medicine, shade, and fencing. In the Tajos of Guanajuato, the people manage 72 species, 47 of which are native such as sage, *garambullo*, and *pitayo*, that they use as food and medicine [98] (Table 2). Mestizo collector groups are also located here, particularly in Aguascalientes, where at least 11 species of the genus *Amaranthus* are collected for food and medicine [99]. Corporate Agriculture threatens these practices by promoting monoculture and substituting food crops for fodder.

**Fig. 9:**
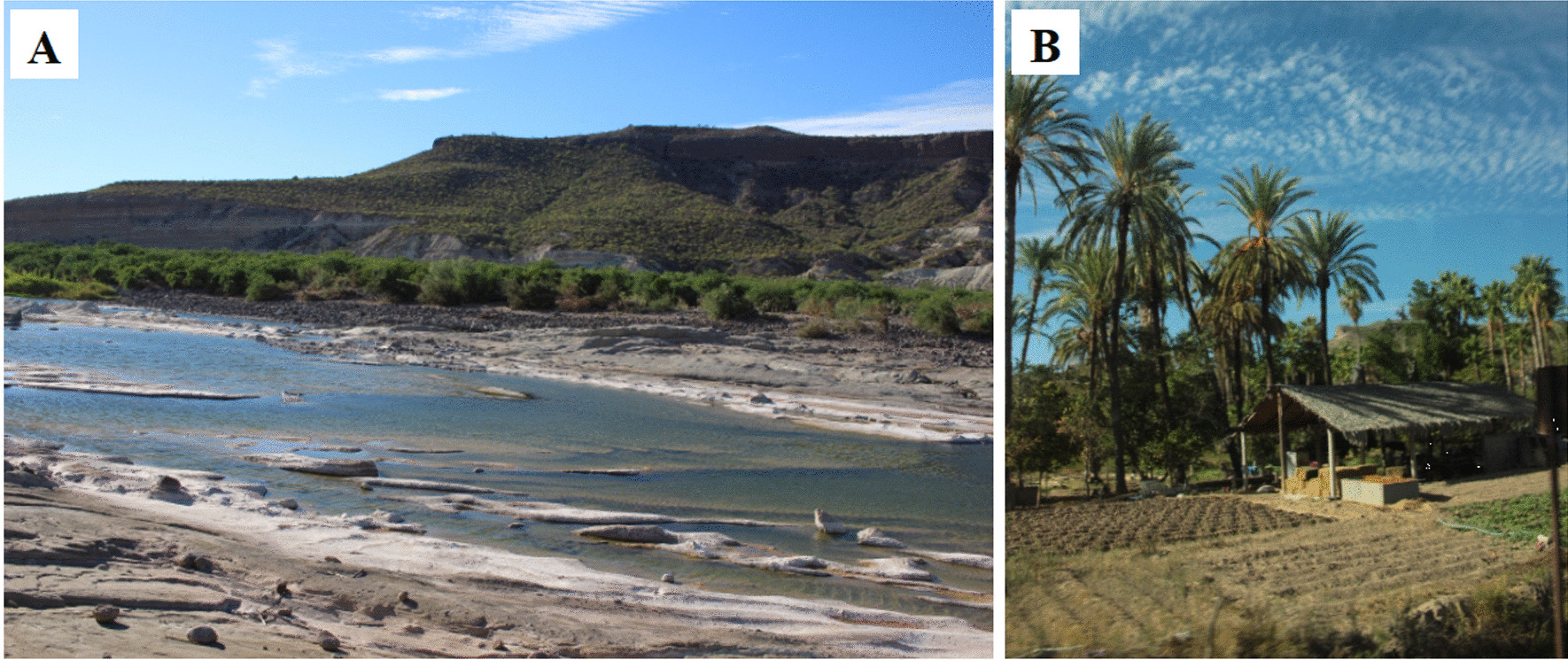
Oases. **A** and **B** Oasis en Baja California Sur, Mexico. Photographs by Enedely Vargas, 2017

*Oases* are a type of colonial agroforestry management and are among the most studied in arid zones (Fig. 9). These ASC develop in natural wetlands in the desert, where cultivated elements inherited from the missionary era interact with fruit trees, livestock, and annual crops. Isolation of these systems contributes to conserving strategies adapted to aridity as complex irrigation systems and stratified crops for water and soil retention.

Cariño et al. [100, p. 152] note that "oases are more than natural wetlands in deserts"; these sites constitute species conservation niches and the relics of management practices imported by the Jesuits from other arid zones in Asia and Europe [16]. The isolation of the Baja California peninsula has allowed the Rancheros, the cultural group finally colonized the region after the expulsion of the Jesuits, to conserve management strategies inherited from the missionary era for centuries. In Baja California Sur, The Rancheros cultivate up to 42 species in multiple strata with water retention and conduction management typical of oases, such as missionary olive, missionary grape, and quince [16] (Table 2).

In Quitovac, Sonora, where Pápagos (Tohono O'odham) survive, up to 139 species are in oasis plots: date palm, fig, lemon, orange, peach, and pineapple, among others [101]. The oases make a significant contribution to the food sovereignty of their inhabitants; they also constitute a relict of conservation of management adapted to and respectful of a fragile environmental balance that does not tolerate overexploitation. However, this fragility represents a significant risk in the face of modernizing ideologies and corporate exploitation that have affected the region since the expansion of the Green Revolution at the end of the last century [16, 100].

**Fig. 10:**
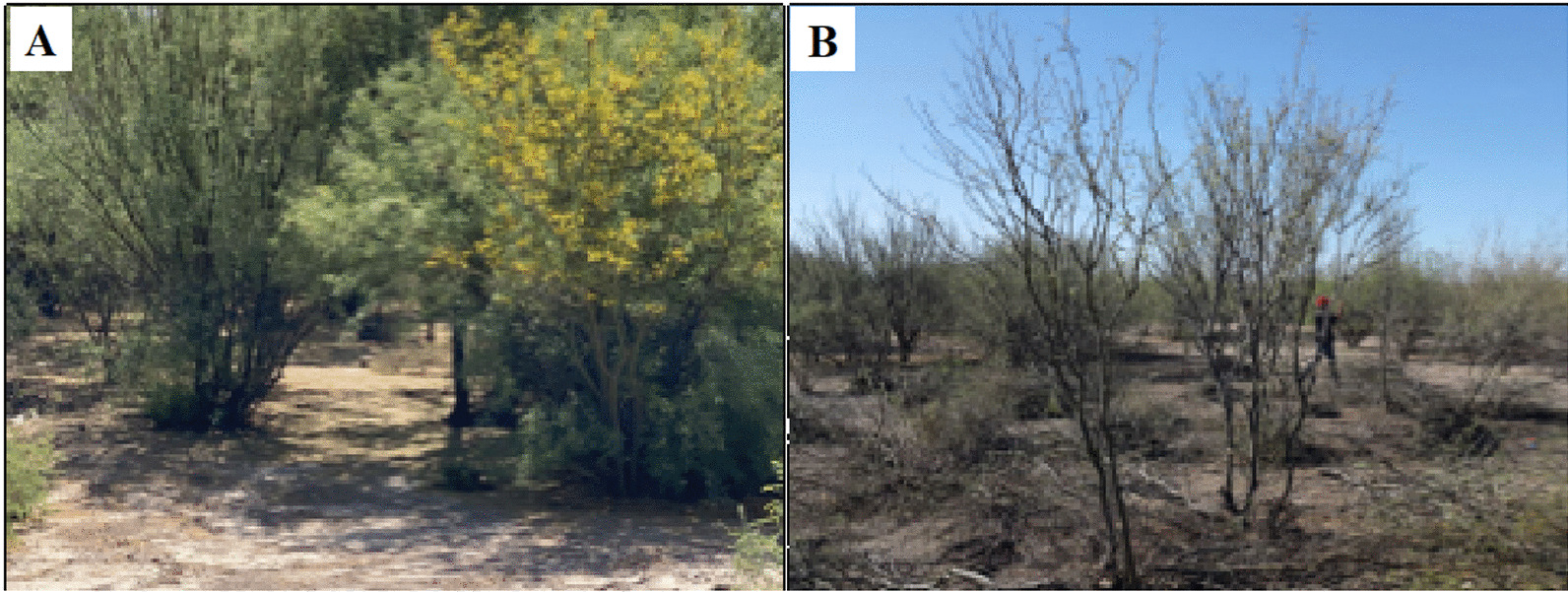
Mesquite and Huisache.** A** Mesquite orchards in Matamoros, Coahuila, Mexico. April 2023. Photography Ing. Samuel Atahualpa Ramírez Macías. **B** Pruning in mesquite orchards in Los Whiles, San Pedro de las Colonias, Coahuila, Mexico. December 2015. Photograph by Alejandro Moreno-Reséndez

The Mesquite and Huisache types represent initiatives to reconnect with the trees that predominate in the scrublands of arid zones (Fig. 10). The case studies are in Baja California, Sonora, Chihuahua, Coahuila, Durango, Nuevo León, San Luis Potosí, and Guanajuato. People are revaluing the multipurpose function of these trees and other native species (Table 2) as food and fodder, for reforestation, to deal with recurring droughts and famines, for soil conservation and recovery, for medicinal purposes, as fuel, and for the production of furniture and handicrafts.

People use the mesquite *péchita* (pod) to make flour, atole, and bread, an ancestral subsistence activity promoted by international and local organizations in the region. [52]. The Mesquite and huisache pods are fodder for cattle, and their flowers are melliferous [102]. These cases constitute a strategy to face environmental changes and contribute to rural families' food sovereignty. However, agricultural policies that favor corporate management threaten this agroforestry due to competition for water.

The cases grouped as Native stand out for the persistence of practices like hunting, fishing, and gathering of local species as essential activities for the subsistence of native groups of Mexican Arid America. The marginalizing conditions of these groups provoke a loss of ancestral knowledge about native biodiversity and abandonment of nomadism as a subsistence strategy and protecting their lands [103].

Sales-Colín et al. [104] reported that *Makurahue* (Guarijío) people cultivated 45 species in the ancient farming systems: *Mahuechi*, *Verano*, and *Solar*. They registered that the guarijíos gathered 58 species in their lands, too. People cultivated since pre-Hispanic times *macuchi*, a local species of tobacco (Table 2) [105].

In the case of the Raramuri rancherías of Chihuahua, most of their plots are on hillsides, where they grow mainly maize and at least five other species. In their homegardens, they report 12 species of fruit trees for self-consumption and the production of fruit preserves for sale. Hunting is very relevant to the Raramuri: they hunt seven minor and two major species, fish in rivers for six species, and collect 20 wild species (Table 2) [103, 106]. Raramuri people obtained from wild and weedy plants, on average, 21.9% of the annual biomass that they consumed [70].

The Seri (*Comcáac*) are probably the last cultural group today to subsist primarily on fishing and gathering. Felger and Mosser [107] reported in 1976 that the Seri people used at least 75 species for food and approximately 95 more in the traditional pharmacopeia. The incorporation of the ethnic group into modern life has caused the disorganization of their nomadic tradition and the integration of the Western diet into their way of subsistence. Therefore, the Seris people today face the loss of their food wealth and ancestral knowledge about their territory [108]. In 2015, Narchi et al. [109] reported 25 species used as medicine in various preparations, 12 species of marine origin, and 13 terrestrial plants (Table 2).

Threats to these cultures are migration, pollution due to tourism activities in the Tarahumara Sierra, and the water fight in the case of the Guarijíos in Sonora, and all ethnic groups face the loss of ancestral relationship with their lands practiced through the fishing, gathering, and hunting.

## Discussion

This work is a first effort to integrate the forms of agri-silvicultural relations in Mexican Arid America. Our research group contributes to this article with the particularities of ASC in the region and the possible reasons for the information gaps detected in previous investigations [1, 4, 8, 12]. It was necessary to articulate visions from different disciplinary fields: geography, history, economics, agroforestry, ethnoecology, ecology, and archaeology. From this interdisciplinary vision, we highlight ASC types with previous records in the literature for Mesoamerica with particularities related to aridity and the modernization and agro-industrialization processes promoted in the north. We also propose three specific types of ASC in the region: Oasis, Native, Mesquite, and Huizache.

Since the last century, research has documented the management of Oases by indigenous and mestizo groups of Sonora and the Baja California peninsula [101]. These systems seemed isolated and alien to those developed in other parts of the country. However, this study emphasizes that Oases are part of the diversity of strategies to inhabit the aridity in the different gradients occurring in Mexican Arid America. With the grouping of Native systems, we try to highlight the importance of the forms of interaction with biodiversity practiced by the native groups of the region, such as harvesting and fishing, which constitute forms of subsistence and niches of conservation of ancestral knowledge on how to inhabit aridity without degrading it. The Mezquite and Huizache grouping highlights recent practices that revalue these species as food, fodder, and for honey production while promoting the conservation of native animal and plant species. These specific systems, together with the traditional Agri-silvopastoral, Silvopastoral, Homegardens, and Milpa systems that occur in Mexican Arid America, represent alternative forms of management in a region of the country that has been identified as the cradle of modernization and agroindustrial development. This article contributes from the academy to rearticulate these practices to the national map of ASC, to break with the northern imaginary that homogenizes the region [22, 30].

The groupings made are not mutually exclusive; production units classified in one system may share characteristics of another. For example, a family could have a backyard homegarden and agro- or silvopastoral management on their plot and their rangeland; or a family could develop a Milpa focused on family food and then convert it into a pasture to feed their livestock. We based the proposed typology on the interactions recorded at the time and place documented by the authors of inventoried publications; it is mainly a methodological strategy to emphasize some aspects of agri-silvicultures but is not definitive. This paper contributes by showing the gaps and opportunities for research on ASC in the region and the heuristic and epistemological potential of studying human-biodiversity interactions from these perspectives.

ASC are dynamic and diversified; these are its main characteristics and strengths. People develop strategies on traditional knowledge and biocultural innovation [1, 4, 8, 12]. They also incorporate entrepreneurial practices to link to the market and access to governmental programs [83, 90, 92]. As a result of these decisions, they suffer the adverse effects of these practices, such as the degradation and contamination of their lands, the loss of ancestral knowledge, and food sovereignty. For these reasons, it is relevant to understand and map ASC and present it as a universe apart from corporate agriculture that should be treated as a specific actor in government policy.

ASC offer food diversity to the families that practice them, and thanks to this diversity, it also protects native species. In our review, we registered around 900 species of plants, 200 of animals, and ten of fungi, including native, introduced, terrestrial, and marine species [16, 84–86, 89–94, 97–99, 101–107, 109]. These biocultural diversity are mainly incorporated as food and medicines, contributing to the food sovereignty of the families that protect and promote them in their spaces. However, the fragility experienced by ASC due to the exclusion of their traditional productive practices in governmental policies remains [83, 87, 100, 105, 108]. For example, in the Yaqui region, it is reported that backyard animal husbandry is not allowed due to sanitary restrictions [93]. In the Tarahumara region, tourism activities threatened these environments [106].

The project of modernization, through agriculturalization and cattle ranching, continues in Mexican Arid America despite the environmental evidence of unsustainability that it is leaving behind, such as the drying up of the valley of Cuatro Ciénegas, Coahuila [80]; the salinity problems in the Santo Domingo Valley in Comondú, Baja California Sur [81], the salinity in the coastal aquifers of Sonora [110]; and the social consequences, such as the struggle for water led by the Yaquis and Guarijíos in Sonora [111]. In contrast, ASC develop adaptation practices such as rainwater harvesting and gray water reuse [95]; nomadisms that respect the natural cycles of native forage recovery [88]; and water management and soil and biodiversity conservation [100, 101, 106].

For academia, the task of continuing to document the practices and interactions of ASC that contribute strategies to strengthen food sovereignty and face environmental change in Mexican Arid America is still pending. The participation of northern academics that research these ASC in research networks established for this purpose [19], or the creation of new research networks specific to the region, is a strategy that should be strengthened or promoted. In this way, it will be possible to build new imaginaries about the agrosilvicultures of the north.

## Conclusions

The reflection carried out in this article aims to show how the Spanish invasion, subsequently the modernizing project headed by the Green Revolution, blurred the diversity of biocultural practices that characterized Mexican Arid America since pre-Hispanic times. From more humid and urbanized perspectives, the knowledge of cultures adapted to the arid and semiarid lands of the north was despised and combated. Modernization tried, like the missionaries and colonizers, to impose a dynamic of agricultural overexploitation on natures other than Mesoamerican ones. This path to modernity, traced without bifurcations and based on ethnocentrisms, generated exclusions of the agri-silvicultures. However, the review results show that the links with biodiversity persist in the native cultures and make their way into other initiatives to reconnect with the wild.

Global changes urge us to stop our civilizing career. They are the niche for reflection to rethink the productivist, extractivist, progressive, and delocalized visions. In academia, these reflections should lead us to stop seeing the desert as a place of "there is no" [22] and not use terms such as "desertification" as if the desert were the result of a process of deterioration and not an ecosystem as diverse and ecologically important, as the jungle or forests. In the way of inhabiting our spaces, this reflection should lead us to accept the humidity or aridity, to put our feet on the ground, literally, and accept once and for all that "we never been modern" [112] or that we were always linked to the earth.

## Data Availability

Not applicable.

## Abbreviations

ASC: Agri-silvicultures
CONAHCYT: Consejo Nacional de Humanidades Ciencias y Tecnología
ITER: Main Results by Locality from the Population and Housing Census 2020
SI: Spanish Invasion
MA: Mesoamerica
AA: Arid America
PRONADE: Programa Nacional de Desmontes
INIFAP: Instituto Nacional de Investigaciones Forestales, Agrícolas y Pecuarias
INEGI: Instituto Nacional de Estadística y Geografía

## Footnotes

1 Civilizing meant reduction, either through military force or evangelization. The integration of the Indians to the sedentary and Christian life, the religious called "reduction" [54].

2 “Living freely roaming the mountains, as practiced by nomadic or semi-nomadic cultures, seemed to constitute an animal way of life and against human nature.” [55 p65].

3 In Sonora, a group of Yaquis, Pimas, and Ópatas, led by the Ópata leaders, the Tánori brothers, fought in sheaves against the liberals and aligned with the French army, perhaps in a bid to regain indigenous autonomy [25].

4 Large agricultural estate belonging to a single owner.

5 The types of “vegetation with possibilities of opening up to agricultural exploitation were: hydrophilic vegetation (mangrove, popal, tular and reedbed), Palmar, Sabana, Selva (high, medium and low), forests (coniferous, deciduous and oak), Mezquital, Chaparral, Matorral (sub-montane, *crasicaule*, desert-rosetophyllous and desertic-microphyllous) and Zacatal (grassland and zacatonal).” [76, p. 123].

6 *Bacanora* is a traditional Sonoran alcoholic beverage made from *agave angustifolia* Haw.
